# Variation in *Plasmodium falciparum* Histidine-Rich Protein 2 (*Pfhrp2*) and *Plasmodium falciparum* Histidine-Rich Protein 3 (*Pfhrp3*) Gene Deletions in Guyana and Suriname

**DOI:** 10.1371/journal.pone.0126805

**Published:** 2015-05-15

**Authors:** Sheila Akinyi Okoth, Joseph F. Abdallah, Nicolas Ceron, Malti R. Adhin, Javin Chandrabose, Karanchand Krishnalall, Curtis S. Huber, Ira F. Goldman, Alexandre Macedo de Oliveira, John W. Barnwell, Venkatachalam Udhayakumar

**Affiliations:** 1 Malaria Branch, Division of Parasitic Diseases and Malaria, Center for Global Health, Centers for Disease Control and Prevention, Atlanta, Georgia, United States of America; 2 Atlanta Research and Education Foundation, Decatur, Georgia, United States of America; 3 Pan American Health Organization—Guyana, Georgetown, Guyana; 4 Department of Biochemistry, Anton de Kom (ADEK) University of Suriname, Paramaribo, Suriname; 5 Guyana Ministry of Health, Georgetown, Guyana; Quensland University of Technology, AUSTRALIA

## Abstract

Guyana and Suriname have made important progress in reducing the burden of malaria. While both countries use microscopy as the primary tool for clinical diagnosis, malaria rapid diagnostic tests (RDTs) are useful in remote areas of the interior where laboratory support may be limited or unavailable. Recent reports indicate that histidine-rich protein 2 (PfHRP2)-based diagnostic tests specific for detection of *P*. *falciparum* may provide false negative results in some parts of South America due to the emergence of *P*. *falciparum* parasites that lack the *pfhrp2* gene, and thus produce no PfHRP2 antigen. *Pfhrp2* and *pfhrp3* genes were amplified in parasite isolates collected from Guyana and Suriname to determine if there were circulating isolates with deletions in these genes. *Pfhrp3* deletions were monitored because some monoclonal antibodies utilized in PfHRP2-based RDTs cross-react with the PfHRP3 protein. We found that all 97 isolates from Guyana that met the inclusion criteria were both *pfhrp2-* and *pfhrp3*-positive. In Suriname (N = 78), 14% of the samples tested were *pfhrp2*-negative while 4% were *pfhrp3*-negative. Furthermore, analysis of the genomic region proximal to *pfhrp2* and *pfhrp3* revealed that genomic deletions extended to the flanking genes. We also investigated the population substructure of the isolates collected to determine if the parasites that had deletions of *pfhrp2* and *pfhrp3* belonged to any genetic subtypes. Cluster analysis revealed that there was no predominant *P*. *falciparum* population substructure among the isolates from either country, an indication of genetic admixture among the parasite populations. Furthermore, the *pfhrp2*-deleted parasites from Suriname did not appear to share a single, unique genetic background.

## Introduction

Suriname, Guyana, and French Guiana account for relatively high numbers of *Plasmodium falciparum* malaria cases compared to other countries in the Caribbean region [1]. Guyana has a population of over 700,000 people [2], most of whom reside on a narrow coastal strip. Malaria transmission does not occur on the coast but is endemic in the interior tropical rainforest regions of the country, including Barima-Waini, Cuyuni-Mazaruni and Potaro-Siparuni; these areas are popular among immigrant workers due to gold mining and logging opportunities [3]. The number of reported malaria cases due to *P*. *falciparum* has been increasing since 2007 and, by 2012, over 20,000 cases of malaria were reported in Guyana [1,4,5].

Suriname borders Guyana to the west, French Guiana to the east and Brazil to the south. By 2004, the population of Suriname was approximately 500,000 individuals, with about 50% of the population residing in the coastal area in and around the capital city, Paramaribo, and approximately 10% living in the interior tropical rainforest regions [6]. The coastal region is separated from the tropical rainforest interior by a savannah belt [7]. Similar to Guyana, malaria transmission in Suriname occurs in the country’s interior [7]. The Suriname-French Guiana border region, especially along the Marowijne River, is known to have one of the highest annual parasite indices in all of South America [1,8]. Nevertheless, increased success in malaria control efforts has resulted in a significant reduction in the number of overall reported malaria cases in Suriname from 14,000 in 2003 to 126 by 2012 [1,9].

*Anopheles darlingi* is the primary malaria vector in Guyana and Suriname [8,10]. Although *P*. *falciparum* is the predominant malaria-causing species in both Suriname and Guyana, *P*. *vivax* also causes a significant number of malaria infections in both countries. *P*. *falciparum* strains in Guyana and Suriname are chloroquine and sulfadoxine-pyrimethamine (SP) resistant [3,11].

In 2004, Guyana and Suriname introduced artemisinin-combination therapy (ACT) as the first line of treatment for uncomplicated *P*. *falciparum* malaria [3]. The ACT consisted of artemether + lumefantrine (Coartem). In 2007, a single gametocytocidal dose of primaquine was included to supplement Coartem treatment in order to reduce malaria transmission [12].

In Guyana, most malaria diagnoses are made primarily by microscopy. However, malaria rapid diagnostic tests (RDTs) are used in the interior where access to microscopic diagnosis is not available. In Suriname, approximately one-third of the health centers have trained microscopists while the rest rely on malaria rapid diagnostic tests (RDTs) as the primary tool for parasite detection [12]. Even so, RDT results are confirmed by microscopic diagnosis of parasite-infected blood smears that are sent to Paramaribo from countrywide health centers [13]. Given the need for RDT use in remote areas, it is important to make sure that the RDTs employed in these countries are reliable.

Most of the commercially available malaria RDTs employ monoclonal antibodies that recognize histidine-rich protein 2 (PfHRP2), which is a *P*. *falciparum*-specific protein [14]. Some monoclonal antibodies found in PfHRP2-based RDTs can cross-react with the protein’s structural homolog, histidine-rich protein 3 (PfHRP3) [15]. Recently, *pfhrp2* gene deletions were detected in 30–40% of *P*. *falciparum* parasite isolates collected from Peru; these deletions resulted in false-negative malaria RDT results when PfHRP2-based diagnostic tests were used [16,17]. Very low levels of *Pfhrp2*-negative parasite isolates have also been reported recently in Mali [18], Senegal [19], and India [20].

**Fig 1:**
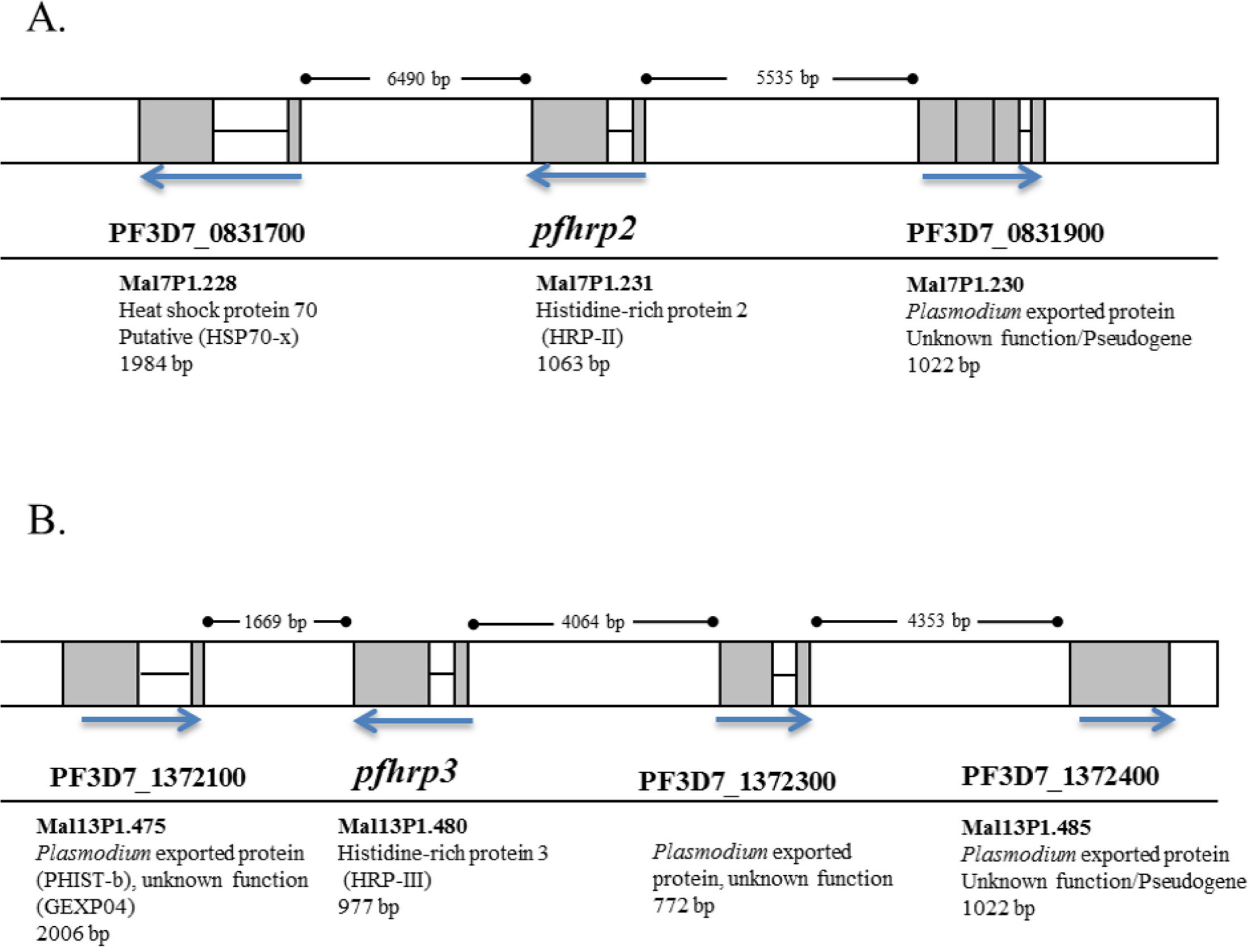
Schematic of the structure of (A) *pfhrp2* (B) *pfhrp3* and their respective neighboring genes. *Pfhrp2* is located subtelomerically on chromosome 8 while *pfhrp3* is located within the non-telomeric region of chromosome 13. The old IDs for each gene (MalxP1.xxx), where available, are indicated below the current PlasmoDB designations. Blue arrows indicate the 5’ to 3’ orientation of each gene. Gene location and information was obtained from PlasmoDB v.9.1 (http://plasmodb.org/plasmo/).

The *pfhrp2* gene (PlasmoDB gene ID: PF3D7_0831800) is 1063 bp long, consists of a single intron and two exons, and is located in the subtelomeric region of chromosome 8 [21–23]. It is immediately flanked by a *Plasmodium* exported protein of unknown function (pseudogene), PF3D7_0831900, and a putative heat shock protein 70 gene, PF3D7_0831700 (Fig 1A). Its structural homolog, *pfhrp3* (PlasmoDB gene ID: PF3D7_1372200) is 977 bp and located subtelomerically on chromosome 13. *Pfhrp3* is immediately flanked upstream by a gene coding for a *Plasmodium* exported protein (PHISTb) of unknown function, PF3D7_1372100 (Fig 1B). A gene coding for acyl-CoA synthetase (PF3D7_1372400) is located approximately 9.1 kb downstream of *pfhrp3* (Fig 1B).

The objective of this study was to determine if there is any evidence for *pfhrp2* and *pfhrp3* deletions in *P*. *falciparum* parasite isolates collected in Guyana and Suriname. We also investigated the population substructure of these *P*. *falciparum* parasite isolates using neutral microsatellite markers with the goal of determining if population substructure has any relationship to the presence or absence of *pfhrp2* and *pfhrp3* genes in this region.

## Materials and Methods

### Ethics Statement

The study protocol for the collection of Guyana blood samples, patient details and travel histories was approved by the ethical review committees of Guyana’s Ministry of Health. The Suriname study was approved by the Institute for Biomedical Science’s (MWI) ethics committee as well as Commissie Mensgebonden Wetenschappelijk Onderzoek, the national ethics committee within Suriname’s Ministry of Health. Written informed consent was obtained from patients or their guardians (if patients were children). The CDC Human Subjects Research office approved CDC investigators to conduct laboratory investigation of the samples from both Guyana and Suriname as this surveillance was determined to be a public health program related activity.

### Sample Collection in Guyana

A total of 100 samples were collected from March to June 2010 (coinciding with the rainy season) as 3 ml venous blood in EDTA tubes. All samples were collected in a single malaria clinic located in Georgetown where most malaria patients from both the coast and interior of Guyana seek treatment. The inclusion criteria for sample collection included positive identification of *P*. *falciparum* infection by microscopy in the blood smears of patients over the age of five, excluding pregnant women. Each blood smear was read independently by two microscopists and if there was a discrepancy in the result, a third expert reader read the slide and confirmed the results. After patients were microscopically confirmed to be positive for *P*. *falciparum*, they were approached to participate in the study. After obtaining written informed consent from patients or their guardians (when patients were children) a venous blood sample was drawn. Blood samples were separated into three aliquots of plasma and three aliquots of packed red cells. In addition, an aliquot of blood was saved on Whatman FTA cards (GE Healthcare, Piscataway, NJ) for parasite specimen preservation. For each sample, one aliquot of packed red blood cells and an aliquot of plasma were provided to the Centers for Disease Control and Prevention (CDC) malaria laboratory for molecular analysis while the rest of the aliquots were stored at the Guyana national reference laboratory for future use. These samples were not tested using RDTs.

### Sample Collection in Suriname

A total of 103 dried blood spot samples collected between 2009 and 2011 that had been saved on Whatman 3MM filter paper ((Whatman, Clifton, NJ, USA) and stored at room temperature in Suriname’s National Malaria Gene Bank were provided to the CDC malaria laboratory for this investigation. Ninety-eight samples were collected at a single malaria clinic located in the capital, Paramaribo, from persons working or living in the interior, while three samples were collected during an Active Case Detection field trip to Benzdorp in Sipaliwini district and two other samples were collected by the Medical Mission in their field clinic located in Tepoe in Sipaliwini district. All patients or their guardians (for children) had provided informed consent for molecular testing upon enrollment and had positive Giemsa-stained thick blood smears for *P*. *falciparum* mono-infection. All malaria-positive slides and 10% of the parasite-negative slides from the Medical Mission (which covers the villages in the interior region of Suriname) were re-evaluated in the laboratory located in Paramaribo as a quality control measure. Only nine samples were tested using Binax NOW RDT test, and the results of the test were confirmed by microscopy.

### Extraction of Parasite DNA and PCR Analysis

Genomic DNA was extracted from either blood or dried filter paper blood spots using the Qiagen QIAamp kit (QIAGEN,Valencia, CA) according to the manufacturer’s instructions. *P*. *falciparum* infection was confirmed by PCR amplification of the *18S* ribosomal RNA gene using methods described by Singh *et al* [24]. We also amplified the merozoite surface protein 2 (*msp2*) gene as previously described to ensure good quality DNA [25]. Only samples for which both *18S rRNA* and *msp2* were successfully amplified were analyzed for *pfhrp2* and *pfhrp3* amplification.

Nested PCR amplifications of *pfhrp2*, *pfhrp3* and their respective flanking genes (Fig 1) were performed using primers and reaction conditions described previously [26]. An *in vitro* cultured parasite isolate, Peru 01–134 (obtained from the Amazon region of Peru) was used as a positive control for all *pfhrp2*, *pfhrp3* and flanking gene experiments. In addition, the laboratory isolate Dd2 was used as a negative control for all *pfhrp2*/flanking genes experiments because this isolate lacks all three genes. Similarly, *in vitro* cultured parasite isolate HB3 was used as the negative control for all *pfhrp3/*neighboring genes experiments because the isolate has deleted all three genes.

PCR amplicons were separated and visualized on a 2% agarose gel. For all PCR experiments, an amplification that resulted in a clearly visible band of the appropriate size was scored as positive for the presence of the appropriate gene. When positive amplification was observed, the result was recorded as final. When there was no amplification of any of the genes tested, then PCR amplification was repeated to confirm this observation. If the result from the second amplification was concordant with the first result indicating no amplification of the gene product, no further testing was done and the result was reported as negative. However, if the second result was discordant with the first, the PCR was performed a third time. The two matching results out of three were scored as the final result.

The prevalence of *pfhrp2-*negative and *pfhrp3*-negative isolates as well as those lacking the flanking genes was calculated by dividing the number of isolates with the specific gene deletion by the total number of isolates determined to be positive for both *18S rRNA* and *msp2* in Guyana (N = 97) and Suriname (N = 78).

### Multilocus Genotyping and Cluster Analysis

Whole-genome amplification was performed on *18s rRNA/msp2*-positive samples using the Repli-G amplification kit (Qiagen, Valencia CA). Seven neutral microsatellite loci were then amplified: TA1 and TA109, both of which are located on chromosome 6; poly α (chromosome 4); PfPK2 (chromosome 12) and 2490 (chromosome 10) [27–31]; C2M34 (chromosome 2) and C3M69 (chromosome 3) [32]. The amplification products were labeled with fluorescent dyes (FAM or HEX) and their sizes assayed on an Applied Biosystems 3130 xl sequencer. The fragments were then scored using GeneMapper software v.3.7 (Applied Biosystems, Foster City CA) with default microsatellite settings, where allele peaks that were lower than 200 relative fluorescence units (rfu) were defined as background. Samples that did not amplify alleles at some loci on the first attempt were re-analyzed to complete the microsatellite profiles.

Only samples containing a single parasite strain (defined as the amplification of only one allele per neutral microsatellite locus) were used for cluster analysis (Guyana N = 29; Suriname N = 57). To determine the population structure of the *P*. *falciparum* isolates, a Bayesian approach was used to infer the number of genetically related clusters (*K*) using the individual neutral microsatellite haplotype profiles. The likelihood of finding between one and ten clusters (*K* = 1 to *K* = 10) was tested for each country’s parasite population using Structure v2.3.3 [33]. In addition, samples from both countries (N = 86) were combined and tested as a single population. We performed twenty replicates of the clustering algorithm for each value of *K* with a burn-in period of 10,000 iterations and 100,000 Markov Chain Monte Carlo replications using the admixture model with correlated allele frequencies [34]. The most likely number of clusters was defined by calculating the Δ*K* value as described by Evanno *et al* [35], where the results from the clustering algorithm in Structure were entered into the Structure Harvester program [http://taylor0.biology.ucla.edu/struct_harvest/][36].

## Results

### Guyana

**Fig 2:**
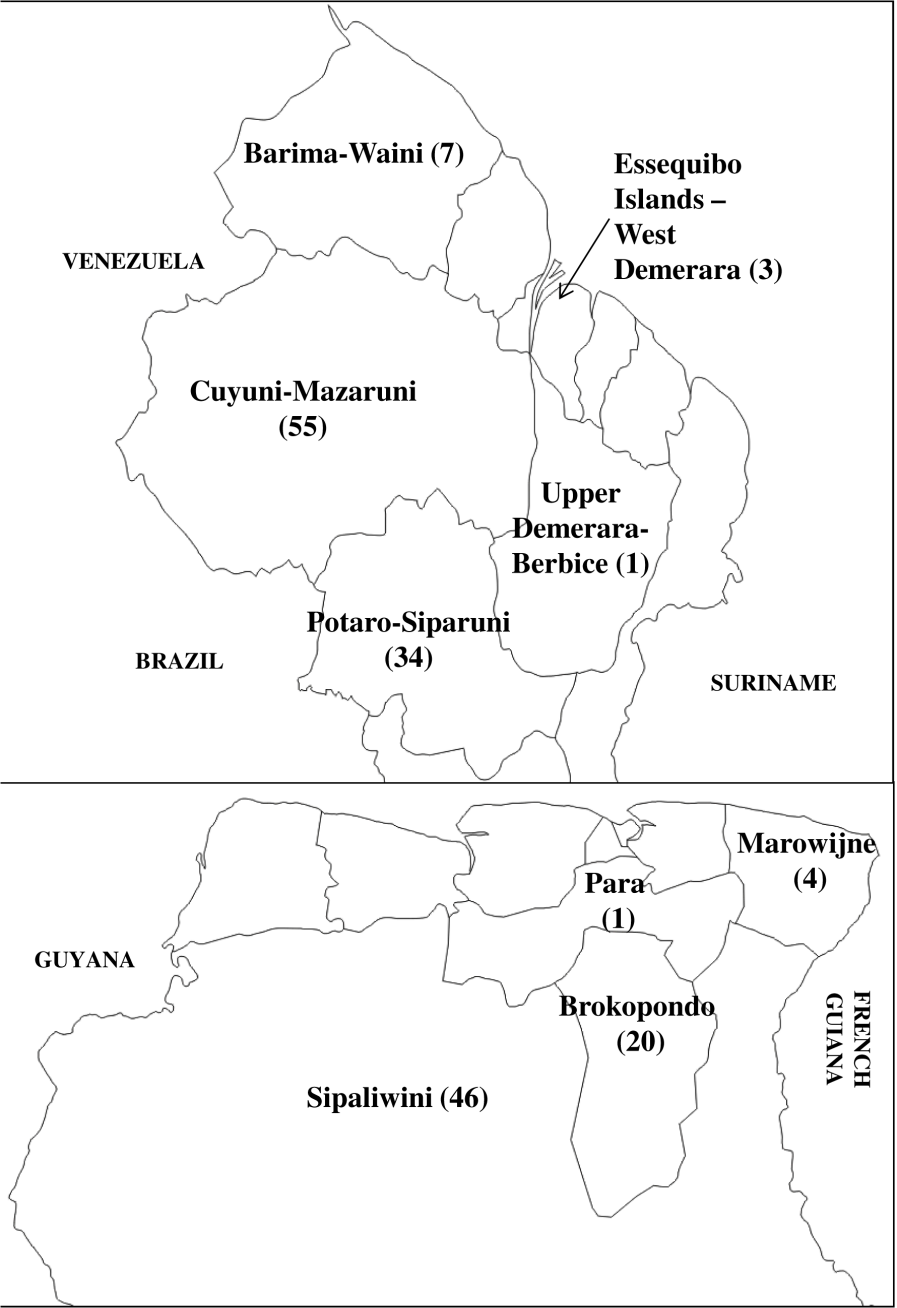
Suspected locations where *P*. *falciparum* infections were acquired based on patient travel histories in regions with malaria transmission two weeks prior to malaria diagnosis in Guyana (Top; total N = 100), and Suriname (Bottom; total N = 78). The number of patients who reported travelling to a particular region (Guyana) or district (Suriname) is indicated in parentheses. The travel history of seven Suriname patients is unknown. Country maps reprinted from d-maps.com under a CC BY license, with permission from Daniel Dalet, original copyright 2007(S1 Supporting Information).

Of the 100 *P*. *falciparum*-infected samples received from Guyana, thirteen were collected from female patients while the rest were from males. All 100 patients reported travelling within the two weeks prior to seeking medical attention for clinical symptoms; the majority had travelled to Cuyuni-Mazaruni (55%) and Potaro-Siparuni (34%) (Fig 2). Three of the 100 samples collected were rejected because they failed to meet the inclusion criterion requiring amplification of both *18S rRNA* and *msp2* genes.

**Fig 3:**
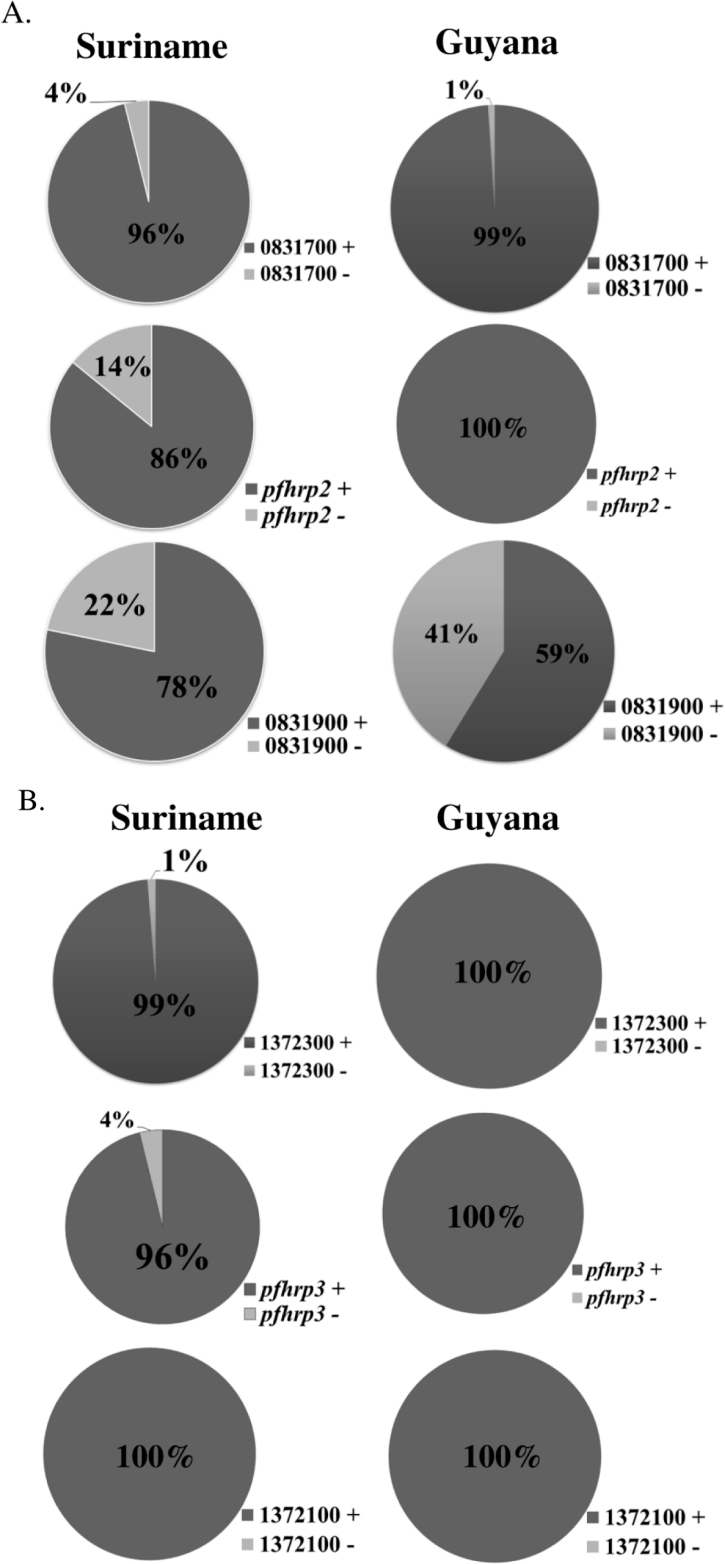
Proportion of deletions in (A) *pfhrp2*, (B) *pfhrp3* and their respective neighboring genes in *P*. *falciparum* isolates collected in Guyana (N = 97) and Suriname (N = 78). The three pie charts to the left of each figure illustrate the proportion of parasite isolates with gene deletions in Suriname samples, while the three pie charts to the right of each Fig show the proportion of isolates with gene deletions in Guyana samples. The percentages shown represent proportions of samples out of the total samples that were *18S RNA*- and *msp-2* positive.

We found that all 97 *18S rRNA*/*msp2*-positive samples from Guyana were positive for both *pfhrp2* and *pfhrp3* genes (Fig 3). However, 40 isolates (41%) had deleted the gene located 5’ of *pfhrp2* (*PF3D7_0831900*) and one sample was negative for the 3’ flanking gene *PF3D7_0831700* (1%; Fig 3A). The genes flanking *pfhrp3* on chromosome 13 were intact in all 97 samples (Fig 3A).

### Suriname

Thirty samples were collected in 2009, 45 in 2010 and 28 in 2011 for a total of 103 specimens. Out of 103 samples, 36 were collected from female patients while 67 were collected from male patients. The majority of patients reported recent travel to Sipaliwini (46%) and Brokopondo (19%) districts. Travel information could not be retrieved for 30 samples. Twenty-five samples were removed from final analysis because they failed to meet the inclusion criterion requiring amplification of both *18S rRNA* and *msp2* genes.

Eleven (14%) of the 78 *18S rRNA*/*msp2*-positive samples from Suriname were *pfhrp2*-negative. Two of the eleven patients from whom the *pfhrp2*-deleted isolates were collected, reported recent travel to Brokopondo district and nine patients had been to Sipaliwini district. All eleven of these *pfhrp2*-negative isolates were collected in 2011; in eight of these samples, exon 2 of *pfhrp2* was intact; three samples had deletions in both exon 1 and 2 of *pfhrp2*. Seventeen of the 78 isolates (22%) had deleted the 5’ *pfhrp2* flanking gene, *PF3D7_0831900*, while three (4%) had deleted the 3’ flanking gene *PF3D7_0831700* (Fig 3B).

We further examined the parasite isolates for gene deletion patterns around *pfhrp2*. Although the majority of samples (67%) were positive for *pfhrp2* and its flanking genes, fourteen isolates (18%) had only deleted the 5’ flanking gene, *PF3D7_0831900* (Table 1A). Other deletion patterns observed included three *pfhrp2*-negative isolates with both flanking genes intact (8%); two *PF3D7_083900*/*Pfhrp2* double-negative isolates (4%); and one *Pfhrp2/PF3D7_0831700*-double negative isolate (3%) (Table 1A).

**Table 1:** Results of PCR amplification of (A) *pfhrp2*, (B) *pfhrp3* and their respective flanking genes in *P*. *falciparum* clinical samples collected in Suriname.

**A**				
**PF3D7_0831900**	***Pfhrp2***	**PF3D7_0831700**	**n**	**%**
+	+	+	52	66.7
-	+	+	14	17.9
+	-	+	6	7.7
-	-	+	3	3.8
+	-	-	2	2.6
+	+	-	1	1.3
**B**				
**PF3D7_1372100**	***Pfhrp3***	**PF3D7_1372400**	**n**	**%**
+	+	+	75	96.1
+	-	+	2	2.6
+	-	-	1	1.3

Only three of the 78 isolates (4%) were *pfhrp3*-negative (Fig 3B). Two of these *pfhrp3*-negative samples were also *pfhrp2*-negative. The gene flanking *pfhrp3* on the 5’ end (*PF3D7_1372100*) was intact in all 78 samples while one sample (1%) was negative for the gene found downstream of *pfhrp3*, *PF3D7_1372400* (Fig 3B).

Lastly, we examined gene deletion patterns around *pfhrp3*. We found that 96% of the 78 isolates from Suriname had intact *pfhrp3* and flanking genes (Table 1B). The following deletion patterns were observed: two (3%) *pfhrp3*-negative samples with intact neighboring genes and one (1%) isolate that was *pfhrp3*/*PF3D7_1372400*-negative but *PF3D7_1372100-*positive (Table 1B).

### Cluster Analysis

Neutral microsatellite genotyping and cluster analysis were performed to investigate a possible relationship among parasite isolates with deletions in *pfhrp2*, *pfhrp3* and/or neighboring genes and their clustering patterns. Structure analysis predicted that there was no identifiable predominant *P*. *falciparum* population sub-structure among the isolates from Guyana or Suriname, even when the two populations were combined and analyzed as a single group (*K* = 1; data not shown).

### Network Analysis

**Fig 4:**
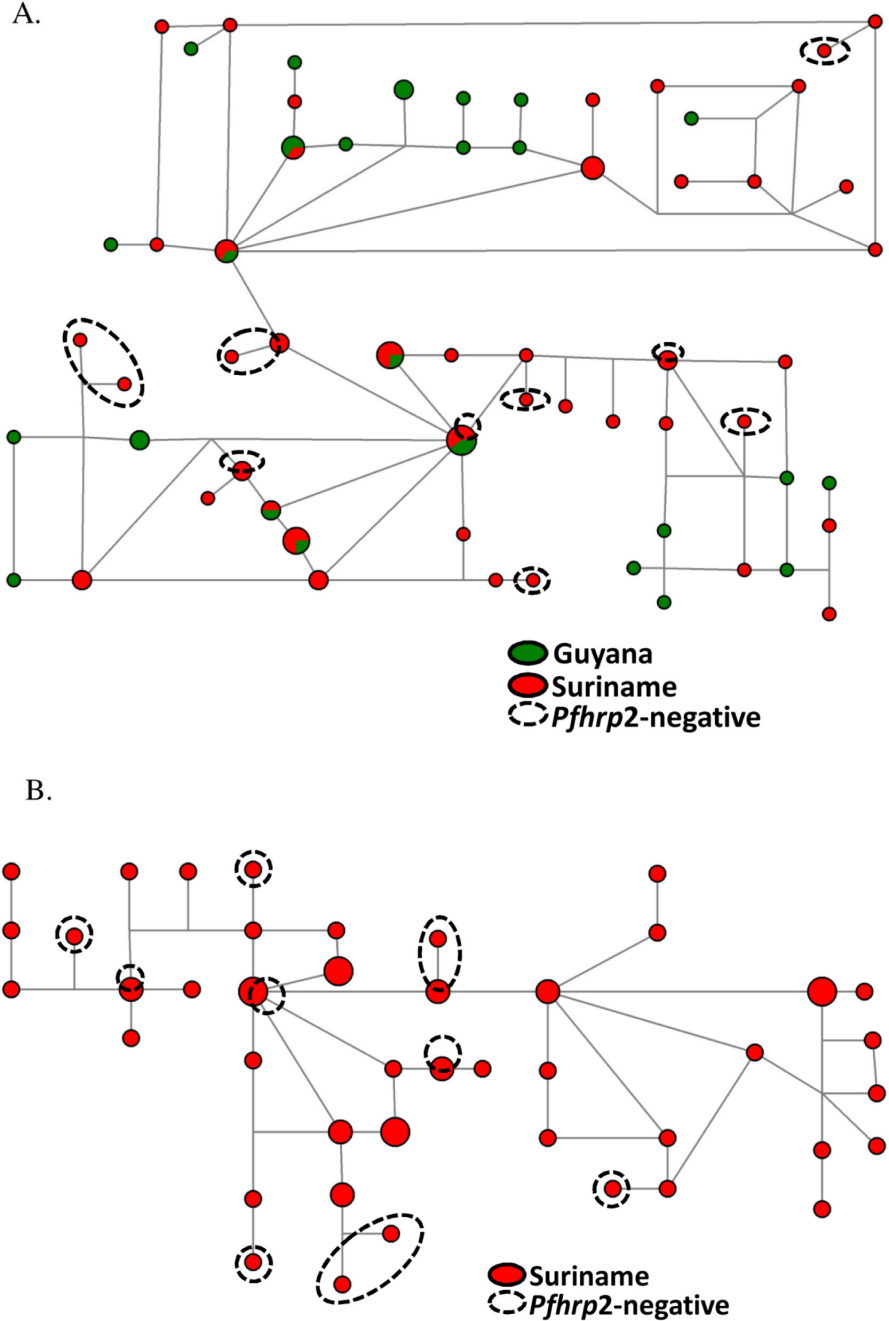
Median joining network analysis of *P*. *falciparum* isolates collected in (A) Guyana (N = 97) and Suriname (N = 57) and (B) Suriname alone (N = 57). The genetic relationships among parasites were constructed using seven neutral microsatellite loci, which have been used previously to genetically characterize *P*. *falciparum* parasite populations in South America. *P*. *falciparum* parasite isolates from Guyana are shown in green while those collected in Suriname are rendered in red. Dotted circles indicate *pfhrp2*-negative isolates.

Median joining network diagrams were created using allele length data at seven neutral microsatellite loci in order to evaluate the genetic relationships among the parasite isolates. No distinct clustering of Suriname isolates separately from those collected in Guyana was observed, indicating that the parasites from the two countries may be very similar genetically (Fig 4A). This outcome confirms the cluster analysis prediction of admixture between the parasite populations from both countries.

The *pfhrp2*-negative isolates, which were collected in Suriname in 2011, did not cluster together as may have been expected, indicating that their genetic backgrounds differed from each other and that they likely did not all originate from a single clonal type (Fig 4B).

## Discussion

Based on genotyping data generated from Guyana isolates, where all samples tested were found to be both *pfhrp2-*positive and *pfhrp3-*positive, it is evident that malaria RDT combo tests that rely on PfHRP2 for the detection of *P*. *falciparum* infections are suitable for continued use in Guyana. This finding is consistent with a recent surveillance study conducted in French Guiana, in which all 221 *P*. *falciparum* isolates collected between 2009 and 2011 were found to be *pfhrp2*-positive; based on this data, it was concluded that PfHRP2-based RDTs were still suitable for use in *P*. *falciparum* malaria diagnosis in French Guiana [37]. In contrast, 14% of the Suriname samples tested had *pfhrp2* deletions while 4% were *pfhrp3*-negative. This is interesting given Suriname’s geographic location between French Guiana and Guyana, where, based on data from this study and others [37], *pfhrp2*-negative isolates were not known to be circulating.

Our data from cluster and median joining network analyses show that *P*. *falciparum* isolates from Guyana and Suriname, although separating into multiple genetic clusters, may be genetically related, probably due to common ancestral relationships and outcrossing among parasites from this region. Furthermore, given that all eleven of the *pfhrp2*-deleted parasite isolates in Suriname were identified in the 2011 collection, but none was found in the two prior years of 2009 and 2010, we sought to determine whether these *pfhrp2*-negative parasites were recently introduced from a single infection. Network analysis of these *pfhrp2*-deleted isolates showed that they belonged to multiple genetic clusters, suggesting that even though these parasites were collected in the same year, they likely did not derive from the clonal expansion of a single *pfhrp2*-negative parasite population. These data are supported by our findings in Peru, where we determined that the evolution and propagation of *pfhrp2*-negative *P*. *falciparum* parasites occurred on multiple genetic backgrounds [17].

Taken together, these data indicate that the occurrence of *pfhrp2*-negative *P*. *falciparum* parasites in Suriname may continue and, therefore, regular monitoring for these parasites is crucial if PfHRP2-based RDTs are considered for use in this country. High proportions of *pfhrp2*-negative *P*. *falciparum* isolates have been reported in Peru, with reported prevalence as high as 40% [16,17]. Given that there were only 331 reported cases of *P*. *falciparum* infections in Suriname in 2011 [1], then the proportion of parasites circulating in Suriname in that year that were *pfhrp2*-negative was approximately 3%. Microscopy remains the primary malaria diagnostic tool in both Suriname and Guyana, but RDTs are used in remote areas where access to laboratory support is limited. Therefore, the presence of *pfhrp2*-negative parasites in Suriname, albeit at relatively low prevalence in 2011, reinforces the need to continue the practice of microscopic confirmation of RDT results in Suriname. In Suriname, it is unclear whether *pfhrp2*-deleted parasites are locally transmitted or whether they are imported by miners travelling from neighboring countries because overall *P*. *falciparum* transmission in the country has significantly decreased. The *pfhrp2* deleted parasites identified in this study were collected from seven male and four female patients who appeared to have been infected while travelling through, or living in, at least two different malaria-endemic districts: Brokopondo, and Sipaliwini. In addition to the indigenous Maroon and Amerindian populations living along the rivers, these two districts attract a very mobile migrant population to the small gold mines scattered across forest areas [12]. It is therefore possible that the *pfhrp2*-negative parasites may have been imported during the migration of laborers looking for work. The two districts vary somewhat geographically: Brokopondo consists mainly of tropical rainforest, is home to the Brokopondo Reservoir and has numerous gold mines, which attract many migrant workers. Sipaliwini, by far the largest (and least populated) district in Suriname, consists of tropical rainforest and borders French Guiana to the east, Guyana to the west and Brazil to the south. The Suriname-French Guiana border region along the Marowijne river is a relatively high malaria transmission region [1]. In spite of the geographic differences among these districts, their commonality in attracting migrant workers to the mines makes them major foci for *P*. *falciparum* (and possibly, *pfhrp2*-negative) parasite transmission. Further studies will be required to determine if the *pfhrp2* deleted parasites found in Suriname are genetically related to those found in other South American countries.

Although *pfhrp2* gene deletion was not found among *P*. *falciparum* isolates collected in Guyana, it is intriguing that 41% of the parasites had deleted the 5’ flanking gene, *PF3D7_0831900* (Fig 3A). 22% of isolates from Suriname also had this deletion (Fig 3B). A recent genome-wide microarray study of *pfhrp2*-negative *P*. *falciparum* isolates from Peru showed that deletion in this genomic locus was not only restricted to *pfhrp2* but encompassed an approximately 20 kb region around the gene [38]. Our previous studies also provided evidence for deletion of the genes proximal to *pfhrp2* and *pfhrp3* in several *P*. *falciparum* isolates, with most of the deletion occurring 5’of each gene [16,17]. Although the mechanism(s) involved and biological significance of these genetic deletions are yet to be elucidated, the prevalence of *PF3D7_0831900-*deleted parasites in Guyana raises the possibility that *pfhrp2*-negative parasites could eventually evolve in this region. Moreover, the migration of mine workers across the borders also raises the possibility that *pfhrp2*-deleted parasites could be introduced into Guyana from elsewhere. Therefore, periodic surveillance for *pfhrp2*-deleted parasites will be necessary if PfHRP2-based RDTs continue to be used in this region.

Some challenges were experienced during the processing of filter paper samples from Suriname. First, almost a quarter of the samples (25 out of 103) had to be excluded from our analyses because of poor quality DNA; these samples did not meet our inclusion criteria of being able to amplify *18s rRNA* and *pfmsp2*. This is possibly because the samples had been spotted on Whatman 3MM filter paper. Furthermore, we experienced challenges in amplifying some genes in a number of samples; in certain instances, gene amplification reactions had to be repeated more than twice. In addition, there were challenges in obtaining travel information for thirty patients, who most likely could not be located due to the constant movement of migrant workers. Lastly, plasma samples were not available to us for serological confirmation of the absence of PfHRP2 protein by ELISA.

It was surprising to find no *pfhrp3*-negative isolates in Guyana and only a limited number of *pfhrp3* deletions in Suriname. In Peru, a larger proportion of parasites was *pfhrp3*-negative compared to those that had deleted *pfhrp2* [16]. However, given the different genomic locations of these genes (*pfhrp2* is located on chromosome 8 while *pfhrp3* is located on chromosome 13), and that the biological significance of these gene deletions is not known, the implications for the apparent differences in proportions of *pfhrp2* versus *pfhrp3* deletions in Suriname compared to Peru is unclear.

In summary, no *pfhrp2* or *pfhrp3* gene deletions occurred in *P*. *falciparum* isolates collected in Guyana. On the other hand, *pfhrp2* gene deletions also did not occur in isolates collected between 2009 and 2010 in Suriname, but were detected in samples collected in 2011. However, it should be noted that a very small number of specimens were collected in 2009 and 2010. The outcome from Suriname illustrates the importance of regular monitoring for *pfhrp2* and *pfhrp3* deletions if PfHRP2-based RDTs continue to be used in the region. Use of non-PfHRP2-based RDTs that target *P*. *falciparum*-specific parasite lactate dehydrogenase (pLDH) with either pan species or *P*. *vivax*-specific pLDH can also be considered as an alternative test for use in Suriname. Furthermore, given that Suriname, Guyana and French Guiana experience influxes of migrant workers, it is likely that *pfhrp2* gene deletions may spread through this mobile population. Therefore, a current sampling of *P*. *falciparum* isolates to update the findings reported here should lead to careful consideration given in choosing appropriate RDTs for use in this region.

## Supporting Information

S1 Supporting Information Permission to publish maps of Guyana and Suriname with modifications. Written permission for the use and modification of the maps in Fig 2 was obtained from Daniel Dalet of d-maps.com.
(PDF)
