# Supplementary material for: Variation in Plasmodium falciparum Histidine-Rich Protein 2 (Pfhrp2) and Plasmodium falciparum Histidine-Rich Protein 3 (Pfhrp3) Gene Deletions in Guyana and Suriname
Source: PLoS One. 2015 May 15;10(5):e0126805. doi: 10.1371/journal.pone.0126805 (PMC4433255; doi:10.1371/journal.pone.0126805)
Supplement: S1 Supporting Information — Written permission for the use and modification of the maps in Fig 2 was obtained from Daniel Dalet of d-maps.com. (PDF) [file pone.0126805.s001.pdf]

Akinyi Okoth , Sheila (CDC/CGH/DPDM) (CTR)

From: Daniel Dalet <daniel.dalet58@gmail.com>
Sent: Friday, March 13, 2015 11:02 AM
To: Akinyi Okoth , Sheila (CDC/CGH/DPDM) (CTR)
Subject: Re: Request for permission to use maps of Suriname and Guyana for scientific publication

Follow Up Flag: Follow up
Flag Status: Flagged

Hi Sheila

Of course I give you this permission, my maps are made for that !

So : I GIVE PERMISSION TO PUBLISH MAPS OF SURINAME AND GUYANA UNDER A CC BY LICENSE TO SHEILA OKOTH

Au revoir !

Daniel Dalet
d-maps.com
Digne (France)

Le 12/03/2015 12:51, Akinyi Okoth , Sheila (CDC/CGH/DPDM) (CTR) a écrit :

Good morning,
I request permission for the open-access journal PLOS ONE to publish the maps of Suriname and Guyana obtained from your website d-maps.com, in a manuscript we are submitting to the journal, under the Creative Commons Attribution License (CCAL) CC BY 3.0 (http://creativecommons.org/licenses/by/3.0/us/). Please be aware that this license allows unrestricted use and distribution, even commercially, by third parties. Please reply and provide explicit written permission to publish the maps of Suriname and Guyana under a CC BY license.

The two maps we are interested in using (with modifications) are found at the following URLs:
Suriname: http://d-maps.com/carte.php?num_car=15549&lang=en
Guyana: http://d-maps.com/carte.php?num_car=15465&lang=en

Sincerely,
Sheila Okoth.

Sheila Akinyi Okoth, Ph.D.
CDC/CGH/ DPDM/Malaria Branch
1600 Clifton Road, Building 23, Room 10/138, MS D-67
Atlanta, GA 30333.
Tel: 404-718-4405.
